# Development of a Tool to Assess the Severity of Pulmonary Hypertension in Patients with Interstitial Lung Disease: A Guide to Assist Therapeutic Choices

**DOI:** 10.3390/arm93050041

**Published:** 2025-10-06

**Authors:** Garrett Fiscus, Chebly Dagher, David O’Sullivan, Brett Carollo, Kristen Swanson, Harrison W. Farber, Raj Parikh

**Affiliations:** 1 Division of Pulmonary, Critical Care and Sleep, Hartford Hospital, Hartford, CT 06106, USA; 2 Division of Internal Medicine, University of Connecticut, Farmington, CT 06030, USA; 3 Department of Research Administration, Hartford HealthCare, Hartford, CT 06106, USA; 4 Division of Pulmonary, Sleep and Critical Care Medicine, Tufts Medical Center, Boston, MA 02111, USA

**Keywords:** pulmonary hypertension, interstitial lung disease, lung transplant

## Abstract

**Highlights:**

**What are the main findings?**

- We created such a scoring tool to guide PH-specific therapy in PH-ILD patients using subjective and objective information (WHO FC, CI, TAPSE, PVR).
- A score of 3 or greater in the PH-ILD Severity score yielded an AUC of 0.831 for the composite endpoint of clinical worsening.

**What is the implication of the main finding?**

- Similarly to the way that risk assessment tools can guide subsequent steps in therapy of PH patients, this PH-ILD Severity score will triage patients who may benefit from inhaled medications, who may require parenteral prostacyclin therapy, and who should be considered for expedited lung transplant evaluation.

**Abstract:**

Background: Pulmonary hypertension (PH) is a frequent complication in patients with interstitial lung disease (ILD); its occurrence results in significant morbidity and mortality. Currently approved treatment options for PH-ILD include inhaled prostacyclin therapy, although this approach may be insufficient in patients who have developed simultaneous right ventricular failure. Moreover, there is no available treatment algorithm regarding the optimal therapy and timing of lung transplant referral for PH-ILD patients based on disease severity. Design/Methods: In this study, we created such a tool to guide PH-specific therapy in PH-ILD patients, especially as further treatment strategies are developed. We developed a 4-point PH-ILD Severity score that integrated both subjective and objective information (WHO FC, CI, TAPSE, PVR) from retrospective analysis of 57 PH-ILD patients. Results: A score of 3 or greater in the PH-ILD Severity score yielded an AUC of 0.831 (*p* < 0.001) for the composite endpoint of clinical worsening (hospitalization due to a cardiopulmonary indication; decrease in 6 min walk distance by >15% at 2 consecutive visits; all-cause mortality; lung transplantation). Conclusions: Further confirmation and evolution of this PH-ILD Severity score will assist in the development of optimal treatment plans in ILD patients diagnosed with concomitant PH.

## 1. Introduction

Pulmonary hypertension (PH) is a condition characterized by increased mean pulmonary artery pressure (mPAP) and pulmonary vascular resistance (PVR) that ultimately leads to right ventricular failure (RVF) and death. PH is classified into five groups based on the causative effect. World Health Organization (WHO) Group 3 PH is a frequent complication in patients with interstitial lung disease (ILD). When diagnosed, PH-ILD is associated with a worse functional status, increased need for supplemental oxygen, and poorer outcomes [1,2,3,4,5,6,7,8].

Until recently, there had been no standard regarding which patients should undergo screening for PH-ILD as there is typically an overlap of symptoms between the two entities. Recently, however, Parikh et al. have developed a PH-ILD Detection tool that strongly suggests concomitant PH in a timely manner, allowing for earlier interventions such as initiation of PH therapy and referral for lung transplant evaluation [5,9]. The clinical benefit of PH-specific therapy in PH-ILD patients was recently demonstrated in the INCREASE trial in which inhaled treprostinil improved 6-min walk distance (6MWD) compared to placebo [10]. Unfortunately, in some patients, PH-ILD can present at an advanced stage with concomitant RV dysfunction, similar to WHO Group 1 pulmonary arterial hypertension (PAH). In such patients, parental prostacyclin therapy may be considered [11,12]. However, while parenteral prostacyclins have well-established benefits in WHO Group 1 PAH, similar outcomes have not been demonstrated in PH-ILD; in addition, these therapies have the potential to worsen hypoxemia by causing ventilation/perfusion (V/Q) mismatch in the setting of parenchymal lung disease [13,14,15].

At present, there is no assessment tool specifically for patients with PH-ILD that can predict disease severity, clinical trajectory, and/or help guide management. To address this gap in PH-ILD management, we have developed a severity score to assist in the treatment approach for PH-ILD patients.

## 2. Methods

We performed a retrospective analysis of 57 PH-ILD patients from a single tertiary academic center. Patients were identified by the International Classification of Diseases 10th Revision (ICD-10) using the diagnosis of PH-ILD from August 2020 to January 2023, who were then followed for one year to assess for clinical worsening [16]. The diagnosis of ILD was confirmed by diffuse parenchymal lung disease on CT chest. The diagnosis of pre-capillary PH was confirmed by right heart catheterization (RHC) with mPAP ≥ 20 mmHg, pulmonary capillary wedge pressure (PCWP) ≤ 15 mmHg, and PVR > 3 Wood units (WU) [17,18]. Other causes of pre-capillary PH, including chronic thromboembolic pulmonary hypertension (CTEPH), along with PH-associated with connective tissue diseases due to the potential for pulmonary arterial hypertension. All 57 patients were initiated PH therapy with either inhaled or parenteral prostacyclin therapy following confirmatory diagnosis via RHC. No other PH therapies were added following the initiation of inhaled/advanced (i.e., subcutaneous, intravenous) therapies. Follow up visits occurred approximately every eight to sixteen weeks following medication initiation. This study was approved by the Hartford HealthCare Institutional Review Board (HHC-2022-0014).

### 2.1. Development of PH-ILD Severity Score

We developed a PH-ILD Severity score that integrated both subjective and objective information: exercise capacity and symptoms from WHO functional class (FC), RHC hemodynamics including Fick-derived cardiac index (CI) and PVR, and echocardiogram (TTE) measurement of tricuspid annular plane systolic excursion (TAPSE) (Table 1). Data were collected prior to initiation of any pulmonary vasodilators. The justification for the inclusion of each of these specific parameters is detailed in the Discussion.

When evaluating these four metrics for the development of the PH-ILD Severity score, several computations were analyzed. The final severity score, ranging from 0 to 4, with each parameter receiving 1 point, performed the best when evaluating for the composite variable of clinical worsening in comparison to the other two versions of the severity score with an area-under-the-curve (AUC) of 0.835 (Table 2). We then evaluated the most appropriate cut-off within the PH-ILD Severity score; this was found to be ≥ 3 based on an AUC of 0.831 (*p* < 0.001).

### 2.2. Clinical Worsening in PH-ILD

The composite endpoint of clinical worsening was utilized as a means for assessing the utility of the PH-ILD Severity score, which was adapted from the INCREASE trial [10]. Clinical worsening was defined as any of the following events: (1) hospitalization due to a cardiopulmonary indication, (2) decrease in 6 min walk distance (6MWD) by >15% at 2 consecutive visits when compared to 6MWD at time of initial PH-ILD diagnosis, (3) all-cause mortality, and (4) lung transplantation [10,17]. Each variable was equally weighted in terms of composite worsening. Although lung transplantation was considered in the clinical worsening composite variable, none of the 57 patients underwent transplantation during the time frame of the study.

### 2.3. Statistical Analysis

A receiver operating characteristics (ROC) curve was generated, and an AUC was calculated from the values of sensitivity (SN) and specificity (SP). All statistical analyses were performed with SPSS v. 29 (IBM, Armonk, NY, USA, 2022), using an a priori alpha level of 0.05.

## 3. Results

### 3.1. Patient Demographics

Patient demographics, baseline clinical data, and subtypes of ILD are shown in Table 3. Within the entire cohort, the mean age was 71 years and there was a male predominance (54%). The most common subtype of ILD was idiopathic pulmonary fibrosis (IPF; 47%), followed by combined pulmonary fibrosis and emphysema (CPFE; 21%). There was a statistically significant difference for DLCO but not for use of supplemental oxygen between the low-risk and high-risk groups as determined by the PH-ILD Severity score. Five patients (17.9%) in the low-risk group were treated with parenteral prostacyclin therapy compared to 15 (51.7%) in the high-risk group (*p* = 0.012).

Of the 12 CPFE patients included in the cohort, 10 patients met the composite endpoint of clinical worsening. There was a heightened concern for adverse effects in response to parenteral prostacyclin in this subtype of ILD; instead, it was noted that 7 of these 10 patients were receiving inhaled therapy at the time of clinical worsening. There were no significant V/Q mismatching that occurred during the year-long follow up.

### 3.2. PH-ILD Severity Score and Parameters

In the low-risk group, patients categorized as WHO FC 2–4 each accounted for approximately one-third of the cohort; however, in the high-risk group, almost two-thirds of patients were categorized as FC 4. Table 4 shows that in the high-risk group, there were significant differences in PVR (*p* < 0.001), CI (*p* < 0.001) and TAPSE (*p* < 0.001) compared to the low-risk group.

### 3.3. PH-ILD Severity Score and Clinical Worsening

**Figure 1:**
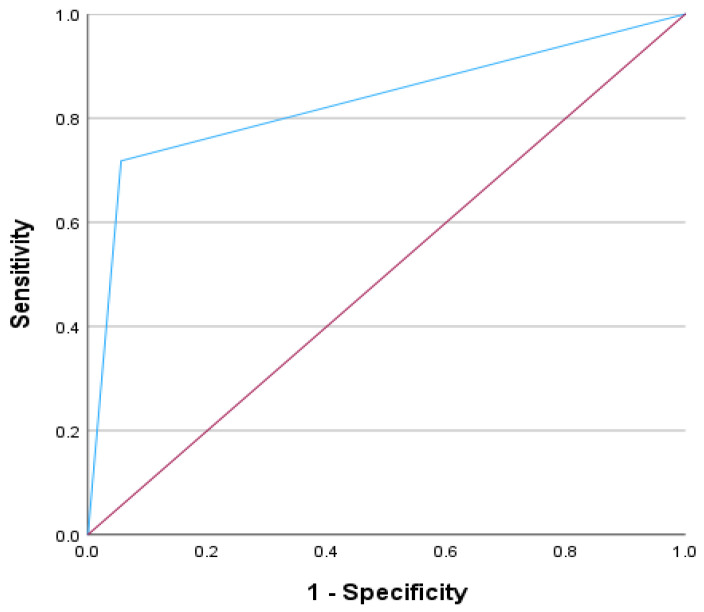
PH-ILD Severity Score and AUC for Clinical Worsening.

A score of ≥3 on the PH-ILD Severity score yielded an AUC of 0.831 (*p* < 0.001) for the composite endpoint of clinical worsening (Figure 1).

## 4. Discussion

PH is an entity that carries significant morbidity and mortality eventually progressing to RVF and death if left untreated [18,19,20,21]. In PAH, utilizing routine risk assessment tools in management has become standard of care, as it not only allows clinicians a consistent method to follow patients longitudinally, but also informs on therapeutic decision-making, including medication titration and referral for lung transplant evaluation. Early recognition of concomitant PH remains a challenge in PH-ILD patients, and the diagnosis may not be established until the disease has progressed to RV dysfunction [5,22,23]. Despite the findings from the INCREASE trial, PH-ILD management is much more complex than a single medication treatment algorithm, especially in the patient who has already progressed to significant RV dysfunction [11,12,17]. Understanding the nuance of the disease and its overall severity and progression, including an evaluation of concomitant RV dysfunction, is paramount to the PH-ILD management approach including evaluation for lung transplant. More importantly, such a tool will certainly become more useful and necessary as additional therapies for ILD are developed and PH-ILD treatment options expand.

### 4.1. Justification of the PH-ILD Severity Score Variables

Developing a severity assessment tool that incorporates both subjective and objective variables while also focusing on parameters that assess RV decompensation was the priority in creating the PH-ILD Severity score. To accomplish this, we extrapolated information from well-established risk assessment tools such as REVEAL and European Society of Cardiology (ESC)/European Respiratory Society (ERS) in order to develop the metrics that were included in this initial PH-ILD Severity score. This iteration of the PH-ILD Severity score included four variables, each allotted 1 point. If the composite score was ≥ 3, the patient was categorized as high-risk PH-ILD since this score was an accurate predictor of the composite endpoint of clinical worsening.

#### 4.1.1. WHO FC

WHO FC is a validated assessment of dyspnea in PH patients [21]. It is widely utilized in disease monitoring and has frequently been employed in PAH clinical trials to assess response to therapy [18,24]. Prior studies have shown a robust association between WHO FC and hemodynamic parameters; since its development, this measurement has been one of the strongest indicators for survival in PAH patients [24,25,26,27,28]. In fact, a worsening WHO FC is considered the most alarming indicator of disease progression [24]. As a result, we incorporated WHO FC ≥ 3 into the PH-ILD Severity score as a validated assessment of a patient’s subjective dyspnea.

#### 4.1.2. PVR

The REVEAL Registry is the largest and most comprehensive registry providing insight into factors that influence survival and prognosis in PAH [29,30]. Similarly to the WHO FC, PVR is included in REVEAL 2.0; a PVR > 5 WU is considered a greater risk for morbidity and mortality [29]. In the INCREASE trial, the average PVR for both treatment and placebo groups was 6.2 WU [17]. Therefore, we established a cut-off of > 5 WU to identify high-risk PH-ILD patients.

#### 4.1.3. Cardiac Index

RVF is a consequence of poorly controlled PH; as such, the ability to improve RV function with PH-specific therapy is an important aspect of disease management [28]. The interventricular septum and ventricular interdependence play an important role in the development of RVF from PH [21,31]. In decompensated PH, RV pressure exceeds left ventricular (LV) pressure causing the interventricular septum to shift leftward leading to the dysfunction of an under-filled LV cavity and subsequent low cardiac output [21]. Thus, CI is an important predictor of survival in PH and as a result, has been included as a staple in several risk assessment scores for PAH [20,21,32]. In PH-ILD, RV dysfunction, reflected by a depressed CI, is believed to play a similar role; therefore, we included CI < 2.0 as a variable in the PH-ILD Severity score to identify patients who should be considered for parenteral prostacyclin therapy in the hope of reversing RV dysfunction [33,34].

#### 4.1.4. TAPSE

Similarly to RHC-derived CI, TTE-derived TAPSE is another method to assess RV function [35,36]. We incorporated a TAPSE < 1.6 cm in the severity assessment of PH-ILD patients since multiple studies have established TAPSE as a reliable prognosticator of poorer outcome in PAH and a marker of significant RV remodeling and dysfunction [18,24,35,36,37,38,39,40,41,42,43,44,45].

### 4.2. Strengths and Limitations

The present study has several limitations including: (1) the data were collected from a single institution, which may introduce data bias; (2) the sample size was relatively small; (3) the parameters were adopted from well-established risk assessment scores that have been evaluated in PAH, but not in PH-ILD; and (4) the severity score relies on TTE-derived TAPSE even though TTE is known to have its limitations in the ILD population [24,45,46,47]. Nonetheless, the development of this severity score and the robust findings from this study introduce a novel concept of PH-ILD risk assessment. Moreover, the tool can and will evolve and will become even more robust as other predictive metrics more specific to PH-ILD are identified and more specific cut points are defined.

## 5. Conclusions

PH-ILD patients have an unacceptably high morbidity and mortality [1,2,3,4,5,6,7]. As such, it is crucial to understand the factors that contribute to this outcome so that current and future therapies, including medical and surgical options, can be considered in a timely manner. While inhaled prostacyclin therapy is now approved for use in PH-ILD, a subset of these patients have severe disease with associated RV dysfunction; in such patients, parenteral prostacyclin therapy may be considered [12]. Similarly to the way that risk assessment tools can guide subsequent steps in therapy of PAH patients, this PH-ILD Severity score will triage patients who may benefit from inhaled medications, patients who may require parenteral prostacyclin therapy and patients who should be considered for expedited lung transplant evaluation. With further validation and evolution as more specific PH-ILD risk metrics and cut-points are identified, such as has occurred with the PAH risk assessment tools, the PH-ILD Severity score will assist in developing optimal treatment plans for ILD patients diagnosed with concomitant PH.

## Figures and Tables

**Table 1:** PH-ILD Severity Score and risk stratification.

Parameter	Score
WHO FC ≥ 3	1
PVR > 5 WU	1
CI < 2.0 L/min	1
TAPSE < 1.6 cm	1
Range 0–4 Low risk: total score < 3 High risk: total score ≥ 3	

**Table 2:** Comparing 3 versions of PH-ILD Severity Score.

Version	WHO FC—pts	PVR > 5	CI < 2	TAPSE < 1.6	Range	AUC	*p* Value
1	3—1 4—2	1	1	1	0–5	0.811	<0.001
2	≥3—1	1	1	1	0–4	0.835	<0.001
3	4—1	1	1	1	0–4	0.823	<0.001

**Table 3:** Baseline characteristics of PH-ILD patients.

Characteristic	Total	Low Risk (≤2)	High Risk (≥3)	*p* Value
Sample size (n, % of sample)	57	28 (49.1)	29 (50.9)	---
Age, years (mean ± SD)	70.9 ± 8.6	70.8 ± 9.7	70.9 ± 7.7	0.974 ^A^
Gender (n, % of category)				0.599 ^B^
Male	31 (54.4)	14 (50.0)	17 (58.6)	
Female	26 (45.6)	14 (50.0)	12 (41.4)	
ILD type (n, %)				0.328 ^C^
IPF	27 (47.4)	13 (46.4)	14 (48.3)	
CPFE	12 (21.1)	4 (14.3)	8 (27.6)	
NSIP	11 (19.3)	7 (25.0)	4 (13.8)	
Sarcoidosis	2 (3.5)	2 (7.1)	0 (0)	
Chronic HP	1 (1.8)	1 (3.6)	0 (0)	
COP	1 (1.8)	0 (0)	1 (3.4)	
Drug-induced	1 (1.8)	0 (0)	1 (3.4)	
HP	1 (1.8)	0 (0)	1 (3.4)	
RB-ILD	1 (1.8)	1 (3.6)	0 (0)	
Antifibrotic therapy (n, %)	20 (35.1)	11 (39.3)	9 (31.0)	0.585 ^B^
PH-specific therapy (n, %)				**0.012** ^B^
Inhaled prostacyclin	37 (64.9)	23 (82.1)	14 (48.3)	
Advanced prostacyclin (i.e., IV/ subcutaneous)	20 (35.1)	5 (17.9)	15 (51.7)	
mPA pressure (average ± SD)	39.2 ±	37.24 ±	44.75 ±	
DLCO (mean ± SD)	32.5 ± 10.8	36.2 ± 9.9	29.0 ± 10.5	**0.010** ^A^
Use of supplemental oxygen (n, %)	48 (84.2)	21 (75.0)	27 (93.1)	0.079 ^B^
Clinical worsening (CW; n, %)	39 (68.4)	11 (39.3)	28 (96.6)	**<0.001** ^B^
Components of CW (n, %)				
1-year Mortality	12 (21.1)	3 (10.7)	9 (31.0)	0.103 ^B^
Hospitalization	30 (52.6)	4 (14.3)	26 (89.7)	**<0.001** ^B^
Decrease in 6MWD of >15%	26 (45.6)	8 (28.6)	18 (62.1)	**0.017** ^B^
Lung transplantation	0 (0)	0 (0)	0 (0)	---

Values in **bold** represent statistically significant (*p* < 0.05) differences. ^A^—Student’s t; ^B^—Fisher’s exact; ^C^—chi square.

**Table 4:** PH-ILD Severity Score parameters.

Parameter	Total (n = 57)	Low Risk (≤2, n = 28)	High Risk (≥3, n = 29)
WHO FC (n, %)			
1	2 (3.5)	2 (7.1)	0 (0)
2	9 (15.8)	9 (32.1)	0 (0)
3	20 (35.1)	9 (32.1)	11 (37.9)
4	26 (45.6)	8 (28.6)	18 (62.1)
PVR (mean ± SD)	8.2 ± 3.6	6.0 ± 2.8	10.4 ± 2.8
CI (mean ± SD)	2.2 ± 0.6	2.4 ± 0.6	2.0 ± 0.4
TAPSE (mean ± SD)	1.7 ± 0.4	2.0 ± 0.2	1.4 ± 0.2

Values in **bold** represent statistically significant (*p* < 0.05) differences.

## Data Availability

The original contributions presented in this study are included in the article. Further inquiries can be directed to the corresponding author.

## Abbreviations

6MWD	6 min walk distance
AUC	area under the curve
CI	Fick-derived cardiac index
CO	cardiac output
COMPERA	Comparative, Prospective Registry of Newly Initiated Therapies for Pulmonary Hypertension
COP	cryptogenic organizing pneumonia
CPFE	combined pulmonary fibrosis and emphysema
CTEPH	chronic thromboembolic pulmonary hypertension
DLCO	diffusional capacity of carbon monoxide
FC	functional class
FPHR	French Pulmonary Hypertension Registry
ICD-10	International Classification of Diseases 10th Revision
ILD	interstitial lung disease
IPF	idiopathic pulmonary fibrosis
IV	intravenous
mPAP	mean pulmonary arterial pressure
NSIP	non-specific interstitial pneumonia
NYHA	New York Heart Association
PCWP	pulmonary capillary wedge pressure
PH	pulmonary hypertension
PAH	pulmonary arterial hypertension
PHISS	PH-ILD severity score
PVR	pulmonary vascular resistance
RB-ILD	respiratory bronchiolitis-associated interstitial lung disease
RHC	right heart catheterization
ROC	receiver operating characteristics
RV	right ventricle
RVF	right ventricular failure
SN	sensitivity
SP	specificity
TAPSE	tricuspid annular plane systolic excursion
VQ	ventilation/perfusion
WHO	World Health Organization
WU	Wood unit
